# Approaches to organic sweet potato cultivation: managing nematodes, pests, and soil health with winter cover crops and biopesticides

**DOI:** 10.3389/fpls.2025.1693056

**Published:** 2025-11-20

**Authors:** Claire M. Schloemer, Scott H. Graham, Koon-Hui Wang, Brent S. Sipes, Bisho R. Lawaju, Kathy S. Lawrence

**Affiliations:** 1 Department of Entomology and Plant Pathology, Auburn University, Auburn, AL, United States; 2 Department of Plant and Environmental Protection Sciences, University of Hawaii at Manoa, Honolulu, HI, United States

**Keywords:** entomopathogenic nematodes, integrated nematode management, majestene, nematode community indices, microbial profile, PLFA, root-knot nematode

## Abstract

The growing demand for organic sweet potato production underscores the need for sustainable pest management and soil health strategies. This study evaluated six winter cover crop systems followed by summer sweet potato, with and without biopesticide applications, to manage *Meloidogyne incognita* and insect pests while assessing soil health indicators. Marketable yield was the highest after wheat (20,679 kg/ha), exceeding the fallow treatment by >2,000 kg/ha. Biopesticide use further increased yield (+700 kg/ha), reduced insect damage by 36%–40% (*p* ≤ 0.05), and enhanced crop value by $33/ha. At planting, *M. incognita* densities were similar across treatments, but by midseason, they were the lowest following rye. Wireworm damage did not vary by cover crop, although biopesticides provided significant protection. Cover crops also shaped nematode communities, with crimson clover, wheat, and mixed systems supporting higher structural index values later in the season, while enrichment index and fungi to bacteria ratios remained unchanged. Soil microbial respiration peaked at planting, especially after wheat and the cover crop mix, and microbial biomass increased across all cover crop treatments, with rye supporting the highest growth. Radish and wheat showed trends toward lower *M. incognita* populations and greater economic returns, although the effects were not statistically significant. Canonical correspondence analysis revealed nematode communities, microbial abundance, and soil CO_2_ flux as key drivers of yield. In 2022, yield was negatively associated with *M. incognita* but positively correlated with fungi to bacteria and Gram-positive to Gram-negative bacteria (GP: GN) ratios; by 2023, yield was instead negatively associated with fungivorous nematodes and microbial respiration and positively associated with protozoa biomass and protozoa to bacteria ratios. Overall, combining cover crops with biopesticides improved yield, reduced pest pressure, and enhanced soil biological function, demonstrating a promising strategy for sustainable organic sweet potato production.

## 1 Introduction

Sweet potato (*Ipomoea batatas*) is the seventh most important food crop worldwide (FAO, 2020). Sweet potatoes are mainly grown for their starchy, nutrient-rich roots, which are used for food, animal feed, and biofuel production (Bovell-Benjamin, 2010). In the United States, the Southeast is the main sweet potato-producing region, with Alabama, Louisiana, Mississippi, and North Carolina being the primary production states (USDA, 2023). Sweet potato adaptability to tropical and subtropical regions, coupled with its drought tolerance and ability to thrive in low-fertility soils, makes it well-suited for low-input organic production (Mukhopadhyay et al., 2011). As organic agriculture has gained popularity (Lotter, 2003), sweet potatoes are cultivated on 57% of American organic farms (Greene and Kremen, 2003). Organic farming must rely on ecologically based pest and fertility management to succeed (Greene and Kremen, 2003). The perceived strategies of the organic production model include improved soil health, reduced pesticide usage, ecological harmony, and lower energy input (Lotter, 2003). Region-based and crop-specific organic farming strategies need to be in place for sweet potato farmers in North Carolina, the highest producers of sweet potatoes in the United States.

Unfortunately, organic pesticides or fertilizers are often less effective or more costly than conventional farming inputs for pest and fertility management. Organic farmers frequently cite the “effectiveness of organically allowable inputs and methods” as a key production constraint and emphasize the need for the effective organic management of insect and nematode pests (Walz, 1999). Plant-parasitic nematodes and insect pests pose significant threats to sweet potato crops (George et al., 2024). In the Southeast, the southern root-knot nematode [*Meloidogyne incognita* (Kofoid and White)] is particularly damaging (Kim and Yang, 2019). Infections by *M. incognita* result in root galling, reduced plant vigor, and lower crop yields (Bird, 1974). In conventional production, both fumigant and non-fumigant chemical nematicides are used, but these practices are not compatible with organic farming (Liu and Grabau, 2022). Although crop rotation with peanuts has been recommended for sweet potato nematode management in the Southeast United States (Davis and Webster, 2005), rotating with winter cover crops (Timper et al., 2006) offers another option, especially since organic post-plant nematicide treatments such as Majestene and MeloCon WG are now available.

In the Southern United States, sweet potato weevils (*Cylas formicarius* Fabricius), white grubs (*Phyllophaga* spp.), wireworms (*Condoderus* spp., *Melanotus* spp., and *Heteroderes* spp.), cucumber beetles (*Diabrotica balteata* LeConte and *Diabrotica undecimpunctata* howardi Barber), and sweet potato flea beetles (*Chaetocnema* spp.) co-infest sweet potato and form the WDS complex (Jennings et al., 2019), causing significant yield losses (Ames et al., 1996). Chemical insecticides, such as bifenthrin and phosmet, are effective chemicals in conventional production (Wallingford, 2023; Webb, 2017). Organic growers could switch to bioinsecticides such as the entomopathogenic fungus, *Beauveria bassiana* (Bals.-Criv) Vuill. and entomopathogenic nematodes like *Steinernema feltiae* Filipjev, *Steinernema carpocapsae* Weiser, and *Heterorhabditis bacteriophora* Poinar (Webb, 2017; Groden, 2012; Kaya and Gaugler, 1993) to manage WDS. Entomopathogenic nematodes decreased wireworm populations but failed to reduce wireworm damage on sweet potato roots at harvest (Seal et al., 2020). However, Schalk et al. (1993) found that *S. carpocapsae* significantly reduced damage to sweet potato roots from WDS when applied three times at monthly intervals. Thus, more research is needed to confirm the effect of entomopathogenic nematodes against WDS. *B. bassiana* is a naturally occurring soil-borne fungus that causes white muscardine disease upon contact with insect hosts (Groden, 2012). The fungus has been formulated into several commercial products, including BotaniGard 22WP (Certis Biologicals, Columbia, MD, USA), Mycotrol (Certis Biologicals, Columbia, MD), and Naturalis-L (Fargro, West Sussex, UK) (Groden, 2012). However, its fungal efficacy on WDS in the field has not been fully documented.

Cover crops, such as legumes, brassicas, and grasses, can suppress plant-parasitic nematodes by producing allelopathic compounds, serving as green manure to alter soil microbial communities, adding soil organic matter (Meyer et al., 2007; Paudel et al., 2021), all of which can reshape soil health conditions that potentially could enhance the performance of bioinsecticides or bionematicides in organic farming systems. Additionally, the increased organic inputs from cover crop biomass enhance free-living nematodes, which contribute to nutrient cycling and microbial balance in the soil (Wang et al., 2011; Fan et al., 2025). Similarly, organic inputs from cover crops improve the performance of biopesticides, particularly those derived from microbial sources such as *Bacillus*, *Paecilomyces*, and *Purpureocillium*, which can directly suppress plant-parasitic nematodes through toxin production, parasitism, or competition, while also promoting soil microbial diversity (Berlitz et al., 2014; Kokalis-Burelle et al., 2017). A shift in nematode community structure, favoring free-living over plant-parasitic nematodes, is a strong indicator of improved soil health and ecosystem functioning (McSorley, 1999).

The effects of cover crops and biopesticides on soil health can be evaluated using various bioindicators. Among biological assessments, soil respiration measured by CO_2_ release and phospholipid fatty acid (PLFA) analysis are widely utilized to provide insights into microbial activity, abundance, and community composition (Paudel et al., 2021). CO_2_ release from the soil estimates microbial metabolic activity, suggesting improved soil microbial function and nutrient turnover (Fraser et al., 2016; Moebius-Clune et al., 2016). PLFA analysis provides a profile of microbial community composition by identifying fatty acid biomarkers specific to bacteria, fungi, and other microbial groups (Fan et al., 2017; Mann et al., 2019; Norris et al., 2023). PLFA profiles can be used to identify changes in microbial diversity and functional groups caused by cover crop residues or biopesticide applications. A balanced microbial community, with a higher fungi to bacteria ratio and increased beneficial microbial populations, is associated with healthier, more resilient soils. Together, soil microbial respiration and PLFA provide a comprehensive understanding of how crops and biopesticides influence soil microbial ecology and overall soil health.

The objectives of the current study were to evaluate the effects of winter cover crops and biopesticides on 1) sweet potato yield and quality, 2) the management of *M. incognita* and WDS insect pests, and 3) soil health.

## 2 Materials and methods

### 2.1 Experimental design

Two winter cover crops and summer sweet potato field trials were conducted in a commercial farm near Dobson, NC. These trials represent the capstone study in a series of experiments (Schloemer et al, 2025), following greenhouse and microplot evaluations in which the most effective cover crops and biological products were identified and selected for testing under commercial field conditions. The field soil consisted of a Colvard sandy clay loam soil consisting of 53% sand, 27% silt, and 20% clay, naturally infested with *M. incognita* race 3 in 2021–2022 and 2022–2023 cropping seasons. Prior to sweet potato planting, winter cover crops were planted in the winters of 2021 and 2022. The winter cover crops tested included crimson clover (*Trifolium incarnatum* L.), daikon radish (*Raphanus sativus* var. *longipinnatus* L.), elbon rye (*Secale cereale* L.), wheat (*Triticum aestivum* L.), and a winter cover crop mix consisting of crimson clover, daikon radish, elbon rye, and wheat. A bare fallow treatment was included as a control. Seeds were obtained from Piedmont Fertilizer Company (Opelika, AL, USA). Each field plot was 7.6 m long and 1 m wide, planted with two rows of cover crops separated by a 4.6-m alley between plots. Cover crop treatments were arranged in randomized complete block design (RCBD) with five replications. The winter cover crops were started in October 2021 and 2022 and were planted by hand as a broadcast seeding to the soil surface at recommended rates for each cover crop. The winter cover crops grew over the winter season and were terminated in April 2022 and 2023 each year using a Bush Hog mower, and the ground was prepared for sweet potato planting by tillage and hill formation. At cover crop termination, a 1-m^2^ area was collected and weighed. The plant biomass was placed in paper bags, allowed to dry naturally for 7 days, and re-weighed. Sweet potatoes were planted on 14 June 2022 and 9 June 2023. Slips of ‘Beauregard’ sweet potato were planted in two rows per plot at 0.3-m spacing within a row using a sweet potato transplanter (US Small Farm Equipment Company, Worland, South Dakota, USA).

Beginning at 4 weeks after transplanting, applications of Triple Threat Beneficial Nematodes entomopathogenic nematodes (EPN) (Arbico Organics, Oro Valley, AZ, USA) consisted of *S. feltiae*, *S. carpocapsae*, and *H. bacteriophora* at 124 million infective juveniles (IJs) of each species/ha, and BotaniGard 22 WP (a.i. *B. bassiana* strain GHA; Certis Biologicals, Columbia, MD, USA) at 4.9 kg/ha was applied monthly to one row of each two-row plot using a handheld sprayer for three applications throughout the growing season. Majestene (Profarm, Davis, CA, USA) containing heat-killed *Burkholderia rinojensis* strain A396 cells and spent fermentation media was applied at 18.7 L/ha in the first two applications.

**Figure 1:**
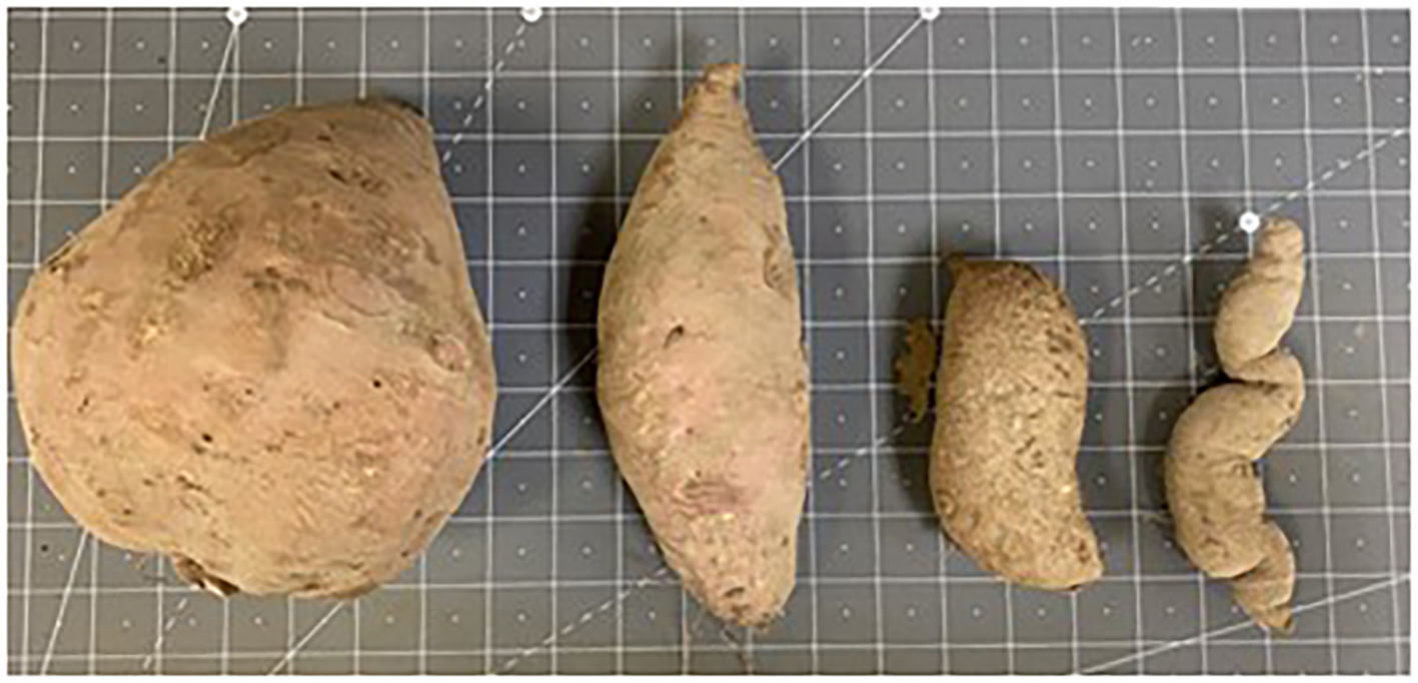
Sweet potato grade classifications of jumbo, No. 1, canner, and cull from left to right.

Sweet potatoes were harvested using a D-10T potato digger (US Small Farm Equipment Co., Worland, WY, USA) on 15 October 2022 (123 DAP) and 14 October 2023 (127 DAP). Sweet potatoes were graded by size and classified as jumbo, No. 1, canner, or cull according to Benedict and Smith (2009), and the number and weight of each grade were recorded for each plot (**Figure 1**). A subsample of No. 1 grade sweet potatoes from each plot was transported to Auburn University’s Plant Science Research Center (PSRC) for insect damage and internal nematode damage assessments. Insect damage was quantified by counting the incidence of the WDS complex (small holes), white grub (large, irregularly shaped holes), and sweet potato flea beetle damage (winding tunnels under the periderm) on five No. 1 grade sweet potatoes/plot (Reed et al., 2009).

### 2.2 Soil data collection

Soil samples were collected at cover crop planting and termination, at sweet potato planting, 30 and 60 days after sweet potato planting, and at sweet potato harvest to monitor soil populations of *M. incognita* and free-living nematodes categorized as bacterivores, fungivores, herbivores, omnivores, and predators. Soil was sampled by collecting 10 cores (2.5 cm diameter × 20 cm deep) from the base of the plants in each plot. The 10 cores were composited in a zippered bag, and all samples were transported to a laboratory. A 100-cm^3^ soil subsample was extracted using a modified gravity sieving and sucrose centrifugation 1.14 sp. G method (Jenkins, 1964). Nematodes were identified to genus and grouped into trophic levels according to their morphology (Goodey, 1963) using a Nikon TSX 100 inverted microscope at ×40–100 magnification. Once the nematodes were identified and quantified, the maturity index (MI), enrichment index (EI), channel index (CI), and structure index (SI) were calculated to monitor soil health and describe the soil food web (Paudel et al., 2021). Soil microbial respiration was also determined at planting and at 30 and 84 DAP using the Solvita CO_2_ Burst test (Woods End Labs, Augusta, ME, USA). Soil samples were dried using a food dehydrator (Excalibur Products, Sacramento, CA, USA) and passed through an 850-µm-pore sieve to remove rocks and other debris. Then, a 30-cm^3^ subsample of each sample was added to the provided internal beaker and interspersed with 9 mL of water through a water dispersion screen. A low-CO_2_ probe was placed into the internal beaker with the moistened soil contained inside a 475-mL Solvita jar and incubated for 24 hours at 20°C. Soil microbial respiration rate was determined by Solvita Digital Color Reader using the CO_2_-Low setting. Soil samples underwent PLFA analysis performed by Regen Ag Lab (Pleasanton, NE, USA).

### 2.3 Data analysis

Data were analyzed using SAS 9.4 (SAS Institute, Cary, NC, USA) using the PROC GLIMMIX procedure. The analysis of variance was conducted with dependent variables including winter cover crop biomass, populations of *M. incognita*, free-living nematode populations, WDS, white grub, flea beetle, total insect damage, sweet potato yields by grade, and soil respiration. The fixed effects were winter cover crop or biopesticide application, and the random effects included replication and years. Student panels were produced to determine the normality of the residuals. There were no significant interactions between replications and years, so these were considered random effects. The Poisson distribution was used for insect damage data. The means of cover crop treatments and biopesticides were separated using the Tukey–Kramer Least Squares (LS)-means test at *p* > 0.10. LS-means presented in the tables, followed by different letters, indicate a significant difference.

Economic analysis was performed by determining the value of sweet potato yields using organic sweet potato prices obtained from a produce packing house. LS-means of economic values were compared between the cover crop treatments and biopesticides using the Tukey–Kramer LS-means test at *p* ≤ 0.10. PLFA analysis was conducted using SAS. Canonical analysis of variance was conducted using Canoco 5.1 (Microcomputer Power, Ithaca, NY, USA).

## 3 Results

### 3.1 Cover crop effects

The highest (*p* ≤ 0.05) cover crop shoot dry weight was recorded on elbon rye (26,404 kg/ha), followed by the winter cover crop mix (**Table 1**). Wheat, crimson clover, and daikon radish supported similar cover crop shoot dry weights. While winter cover crops were actively growing, soil populations of *M. incognita* were similar between all cover crops, although all were higher than the soil threshold level of 10 *M. incognita*/100 cm^3^ soil (Schloemer et al., 2025). At sweet potato planting, the abundance of *M. incognita* was similar in all winter cover crops as the fallow, ranging from 23 to 77 *M. incognita*/100 cm^3^ soil (**Table 1**). Sweet potatoes that followed wheat and fallow had numerically the lowest soil populations of *M. incognita*, but no statistical differences were observed among any of the cover crop treatments at 30 days after the sweet potato planting, and overall *M. incognita* populations had increased an average of 35% from planting. At midseason (60 DAP), *M. incognita* populations had increased 67% similarly across the entire test. At harvest, near 84 DAP, *M. incognita* populations were still similar across all plots.

**Table 1:** Effects of winter cover crops on populations of *Meloidogyne incognita* in the soil throughout the sweet potato 2022 and 2023 cropping seasons.

Winter cover crop	Winter cover crop biomass^a^(kg/ha)		*M. incognita*/100 cm^3^ soil Near cover crop termination		At plant *M. incognita*/100 cm^3^ soil		30 DAP^b^*M. incognita*/100 cm^3^ soil		60 DAP *M. incognita*/100 cm^3^ soil		84 DAP *M. incognita*/100 cm^3^ soil	
Fallow	7,045	d^c^	30	a	15	a	36	a	150	a	134	a
Crimson clover	14,753	cd	46	a	21	a	44	a	116	a	131	a
Daikon radish	10,197	cd	38	a	18	a	44	a	60	a	82	a
Elbon rye	26,404	a	77	a	26	a	77	a	24	a	49	a
Wheat	17,895	bc	31	a	15	a	64	a	81	a	103	a
NC mix	24,403	ab	23	a	21	a	64	a	54	a	225	a
*p*-Value^d^	0.0001****		0.4982		0.948		0.1792		0.664		0.658	

^a^Winter cover crop biomass was assessed as dry weight of aboveground biomass. ^b^Days after planting (DAP). ^c^Values followed by the same letter are not significantly different at *p* ≤ 0.1 as determined using the Tukey–Kramer method. ^d^*p*-Values for Type III fixed effects with significance at the 0.001 levels are indicated by ****.

### 3.2 Nematode community

**Figure 2:**
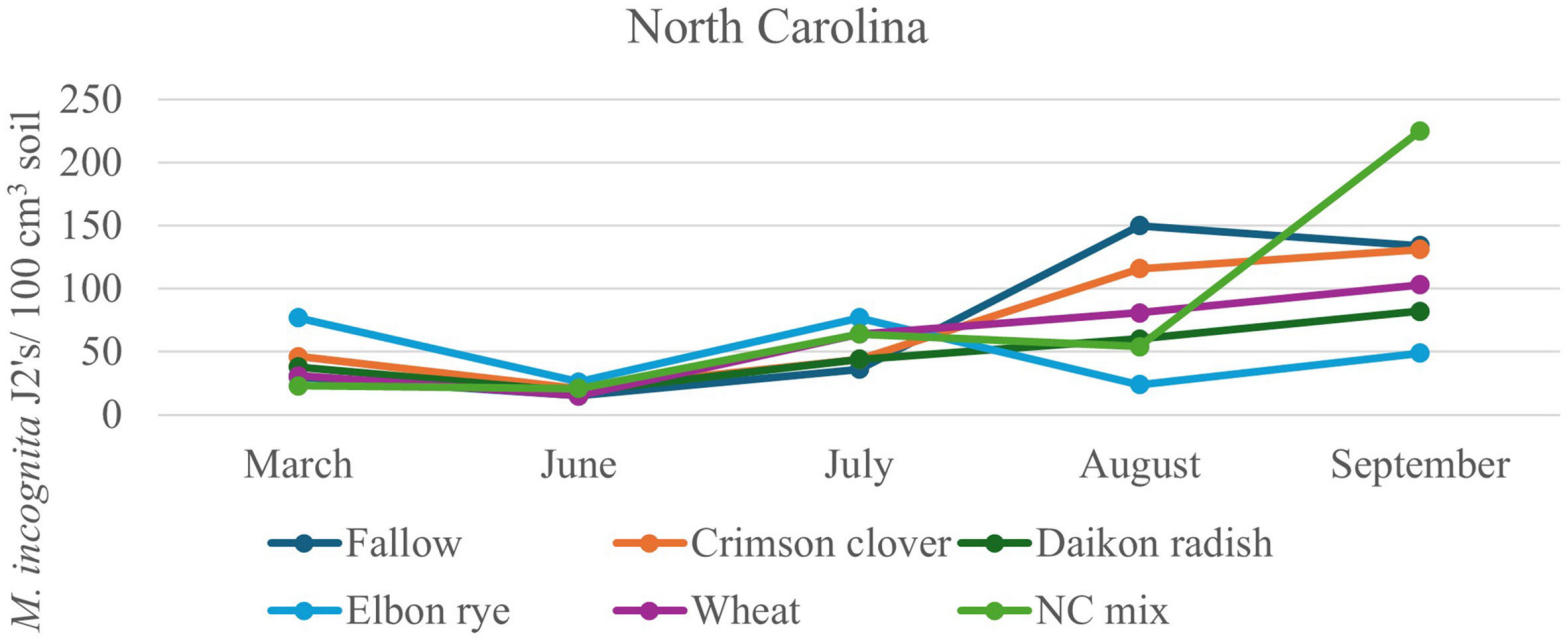
Soil population densities of *Meloidogyne incognita* across the sweet potato cropping season following the winter cover crops in North Carolina, 2022–2023.

**Figure 3:**
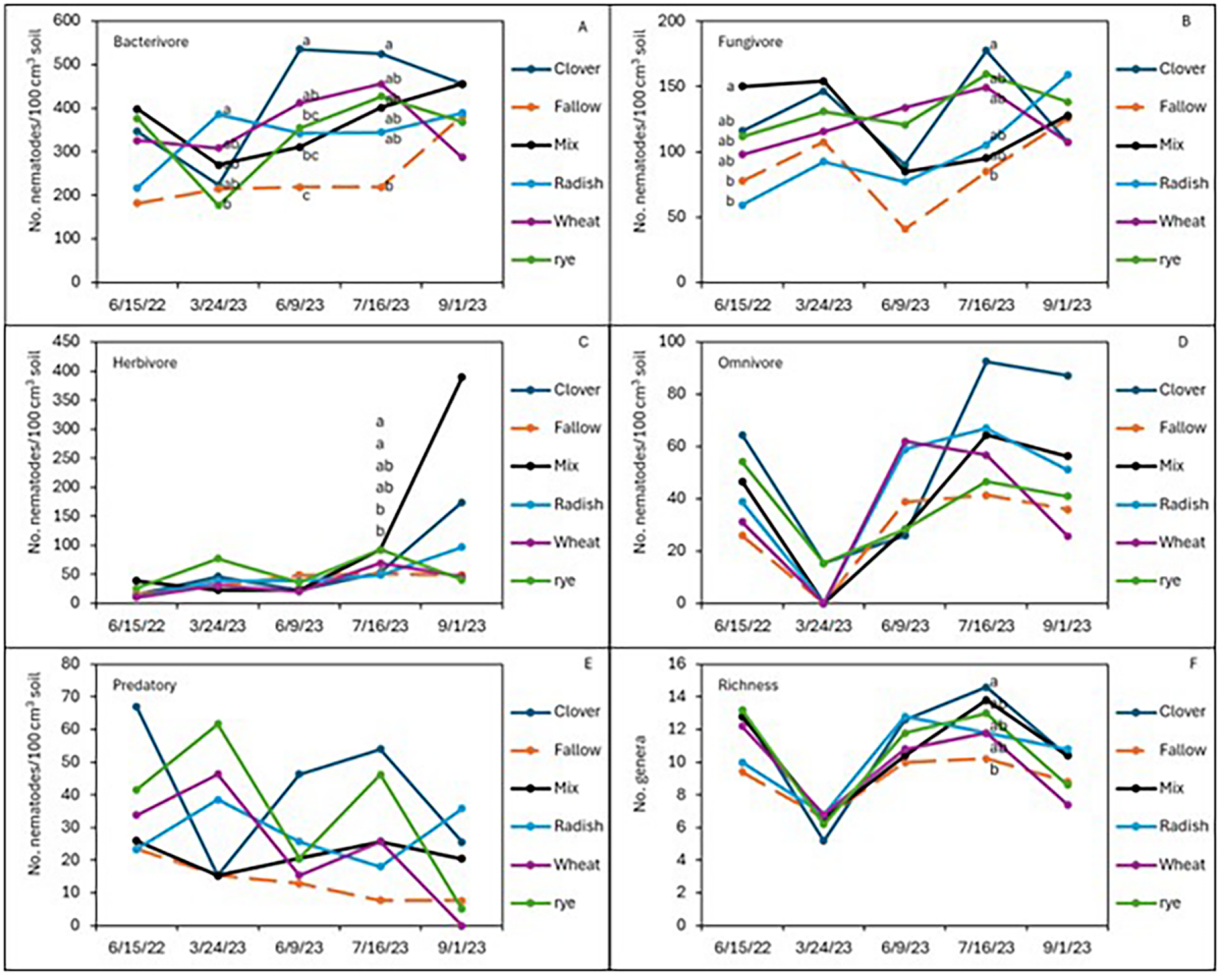
Soil populations of nematode trophic groups: bacterivores **(A)**, fungivores **(B)**, herbivores **(C)**, omnivores **(D)**, and predators **(E)**, along with richness **(F)** during the sweet potato growing season following the winter cover crops in the sweet potato 2022 and 2023 cropping seasons.

After 2 years of winter cover cropping and organic sweet potato production, soil nematode communities were monitored and compared with a baseline sample from spring 2022. Radish increased the abundance of bacterivorous nematodes compared to fallow (*p* < 0.05; **Figure 2A**), but wheat maintained higher numbers of bacterivores in the early season and midseason of sweet potato, while the cover crop mix managed to have similar abundance of bacterivores at harvest as wheat, although there were no significant differences in bacterivores among the treatments at sweet potato harvest. Clover also increased the abundance of fungal-feeding nematodes at the midseason of sweet potato (*p* ≤ 0.05, July 2023) compared to fallow plots (**Figure 2B**). The winter cover crop mix and rye supported higher populations of herbivorous (plant-parasitic) nematodes than fallow at 1 month after sweet potato planting, but this effect was no longer significant at harvest (**Figure 2C**). Although not statistically different, crimson clover had a higher abundance of omnivorous and predatory nematodes (**Figures 2D, E**) and significantly higher nematode richness than the fallow 1 month after sweet potato planting (July 2023; **Figure 2F**). When calculating these nematode data set into nematode community indices (**Figure 3**), despite higher structural index (SI) in the fallow treatment at sweet potato planting, crimson clover, and wheat and cover crop mix resulted in numerically higher SI than fallow, while having no differences in the EI and (Fungivores/Fungivores + Bacterivores) (F/F+B) at harvest (**Figure 3**).

### 3.3 Insect damage

Winter cover crop treatment did not show any effect on WDS complex damage (*p* > 0.05; **Table 2**), with overall low damage ranging from 2.43 to 3.53 small holes/root in crimson clover and fallow, respectively. However, the application of biopesticides significantly (*p* < 0.001) reduced WDS complex damage by 40% compared to the untreated plots. White grub, sweet potato flea beetle, or root-knot nematode (root cracking) damage was too low to detect a difference by the biopesticide combination effect in this location. The addition of biopesticides significantly (*p* < 0.001) reduced total insect damage by 36% (**Table 2**).

**Table 2:** Analysis of variance (ANOVA) of winter cover crop and biopesticide effects on sweet potato root damage due to WDS complex, white grubs, sweet potato flea beetles, and *Meloidogyne incognita* in the two sweet potato cropping seasons 2022 and 2023.

	WDS complex damage^a^		White grub damage^b^		Sweet potato flea beetle damage^c^		*M. incognita* damage^d^		Total insect damage^e^	
Winter cover crop (WCC)	0.3236		0.9132		0.4519		0.6056		0.3512	
Biopesticide	0.0001***		0.4706		0.6931		0.291		0.0001***	
WCC × Biopesticide	0.6979		0.1851		0.4018		0.9508		0.507	
Biopesticide^f^										
Untreated	3.54	a^g^	0.21	a	0.08	a	0.01	a	3.83	a
Treated	2.13	b	0.25	a	0.07	a	0.03	a	2.45	b
*p*-Value^h^	0.0001****		0.4759		0.6933		0.2825		0.0001****	
Winter cover crop										
Fallow	3.53	a	0.17	a	0.13	a	0.03	a	3.83	a
Crimson clover	2.43	a	0.23	a	0.06	a	0	a	2.72	a
Daikon radish	2.53	a	0.27	a	0.08	a	0.03	a	2.88	a
Elbon rye	2.58	a	0.25	a	0.07	a	0.03	a	2.89	a
Wheat	3.21	a	0.27	a	0.04	a	0	a	3.52	a
NC mix	2.72	a	0.19	a	0.07	a	0.05	a	2.98	a
*p*-Value^h^	0.3127		0.9173		0.4526		0.5865		0.3472	

^a^WDS complex consists of wireworm spp., *Diabrotica* spp., and *Systena* spp.; damage was assessed as average number of insect holes per sweet potato. ^b^White grub (*Phyllophaga* spp.) damage consists of wide tunnels gouged into the surface of sweet potato roots. ^c^Sweet potato flea beetle (*Chaetocnema* spp.) damage consists of thin, winding tunnels etched into the sweet potato periderm. ^d^*M. incognita* damage consists of root cracking. ^e^Total insect damage is expressed as the sum of WDS complex, white grub, and flea beetle damage, which was assessed as average number of insect damage incidences per sweet potato. ^f^Biopesticides consisted of Triple Threat Beneficial Nematodes; *Steinernema feltiae*, *S. carpocapsae*, and *Heterorhabditis bacteriophora* at 123.5 million IJ’s/ha, BotaniGard 22 WP; *Beauveria bassiana* strain GHA at 4.9 kg/ha; and Majestene, heat-killed *Burkholderia* spp. strain A396 cells and spent fermentation media 18.7 L/ha. ^g^Values followed by the same letter are not significantly different at *p* ≤ 0.1 as determined using the Tukey–Kramer method. ^h^*p*-Values for Type III fixed effects with significance at the 0.01 and 0.001 levels are indicated by *** and ****, respectively.

### 3.4 Sweet potato yield

No significant interactions in sweet potato yield parameters between the cover crops and biological applications were detected. Thus, the effects of cover crops and biopesticides on sweet potato yield were summarized separately. Cover crops did not significantly affect the number or weight of jumbo, No. 1, or canner grade sweet potatoes across winter cover crops (**Table 3**). However, the winter cover crop mix produced significantly more canner grade sweet potatoes than fallow, with >9,000 sweet potatoes/ha increase. Elbon rye produced the highest yield of sweet potatoes by weight (4,555 kg/ha), with 1,200 kg/ha more than that of the fallow. Total marketable yield that included jumbos, No. 1, and canners was the highest following the wheat winter cover crop (20,679 kg/ha), 2,000 kg/ha higher than the fallow. In contrast, biopesticides did not affect the number or weights of jumbo, No. 1, or canner grade sweet potatoes (*p* > 0.05). Nonetheless, biopesticides numerically increased sweet potato yield by 700 kg/ha more than the untreated plots.

**Table 3:** Sweet potato root numbers, yield (kg/ha), and economic value by quality grade with and without biopesticides following winter cover crops in the two sweet potato cropping seasons 2022 and 2023.

Source of variation (F-value)	Jumbo count^a^(number/ha)		Jumbo weight (kg/ha)		No. 1 count (number/ha)		No. 1 weight (kg/ha)		Canner count (number/ha)		Canner weight (kg/ha)		Total marketable yield^b^(kg/ha)		Value of marketable yield ($/ha)	
Winter cover crop	0.7491		0.5999		0.5687		0.5971		0.0552*		0.0952*		0.8652		0.9315	
Biopesticide^c^	0.8058		0.4553		0.9277		0.7311		0.8832		0.5714		0.4921		0.8255	
Winter cover crop × Biopesticide	0.2757		0.1654		0.9189		0.9375		0.4834		0.6174		0.9149		0.9798	
Biopesticide																
Untreated	3,707	a^d^	3,245	a	37,358	a	11,720	a	33,627	a	3,947	a	18,913	a	$1,781	a
Treated	3,827	a	3,575	a	37,167	a	11,972	a	33,914	a	4,113	a	19,659	a	$1,814	a
*p*-Value^e^	0.807		0.4613		0.9621		0.8193		0.8867		0.5864		0.5597		0.8161	
Winter cover crop																
Fallow	3,803	a	3,366	a	37,669	a	11,764	a	28,557	b	3,289	a	18,419	a	$1,793	a
Crimson clover	4,520	a	4,180	a	34,225	a	10,978	a	33,149	ab	3,717	a	18,875	a	$1,709	a
Daikon radish	3,516	a	3,343	a	37,238	a	12,517	a	31,714	ab	3,851	a	19,711	a	$1,864	a
Elbon rye	3,229	a	2,760	a	38,601	a	11,926	a	37,597	ab	4,555	a	19,241	a	$1,800	a
Wheat	3,875	a	3,547	a	40,539	a	12,870	a	33,651	ab	4,263	a	20,679	a	$1,930	a
NC mix	3,659	a	3,265	a	35,301	a	11,021	a	37,956	a	4,503	a	18,789	a	$1,688	a
*p*-Value^e^	0.7544		0.6144		0.9562		0.8965		0.0693*		0.1219		0.9289		0.9257	

^a^Yield was separated by quality size classification into jumbo, No. 1, and canner grades. ^b^Total marketable yield is expressed as the sum of the weights of jumbos, No. 1, and canners. ^c^Biopesticides consisted of Triple Threat Beneficial Nematodes; *Steinernema feltiae*, *S. carpocapsae*, and *Heterorhabditis bacteriophora* at 123.5 million IJ’s/ha, BotaniGard 22 WP; *Beauveria bassiana* strain GHA at 4.9 kg/ha; and Majestene, heat-killed *Burkholderia* spp. strain A396 cells and spent fermentation media 18.7 L/ha. ^d^Values followed by the same letter are not significantly different at *p* ≤ 0.1 as determined using the Tukey–Kramer method. ^e^*p*-Values for Type III fixed effects with significance at the 0.1 level are indicated by *.

In terms of economic values, although the economic value of the sweet potato crops was similar between the biopesticide and untreated control (*p* > 0.05), the biopesticides increased the crop value by $33/ha. All the cover crops also supported similar sweet potato yields, although wheat and daikon radish increased the value of sweet potato by $137 and $71/ha, respectively.

### 3.5 Soil health

The microbial respiration values were high at sweet potato planting. The highest value was recorded on the winter cover crop mix, followed by wheat, although no statistical differences were seen across winter cover crops at sweet potato planting (**Table 4**). At 30 DAP, the highest microbial CO_2_ release occurred following crimson clover and the winter cover crop mix, with values of 96.5 and 95.3 ppm, respectively, which was 27% higher than the lowest microbial CO_2_ release occurring following the elbon rye. No statistical differences were measured between the different cover crops at 84 DAP, but the highest values of 95.2 ppm CO_2_ were recorded following the wheat and the winter cover crop mix.

**Table 4:** Soil CO2 release as measured by a Solvita CO2 burst during the sweet potato cropping season following the winter cover crops in 2022–2023.

Winter cover crop	At plant soil CO_2_ release^a^(ppm)		30 DAP^b^soil CO_2_ release (ppm)		84 DAP soil CO_2_ release (ppm)	
Winter cover crop						
Fallow	57.4	A^c^	79.2	ab	84.5	A
Crimson clover	54.6	a	96.5	a	88.1	A
Daikon radish	56	a	78.6	ab	76.3	A
Elbon rye	61.5	a	69.6	b	77.6	A
Wheat	65.5	a	84.1	ab	95.2	A
NC mix	69.2	a	95.3	a	95.2	a
*p*-Value^d^	0.892		0.0258**		0.231	

^a^Soil CO_2_ respiration was measured using the Solvita CO_2_ Burst procedure. ^b^Days after planting (DAP). ^c^Values followed by the same letter are not significantly different at *p* ≤ 0.1 as determined using the Tukey–Kramer method. ^d^*p*-Values for Type III fixed effects with significance at the 0.1, 0.05, 0.01, and 0.001 levels are indicated by *, **, ***, and ****, respectively.

**Figure 4:**
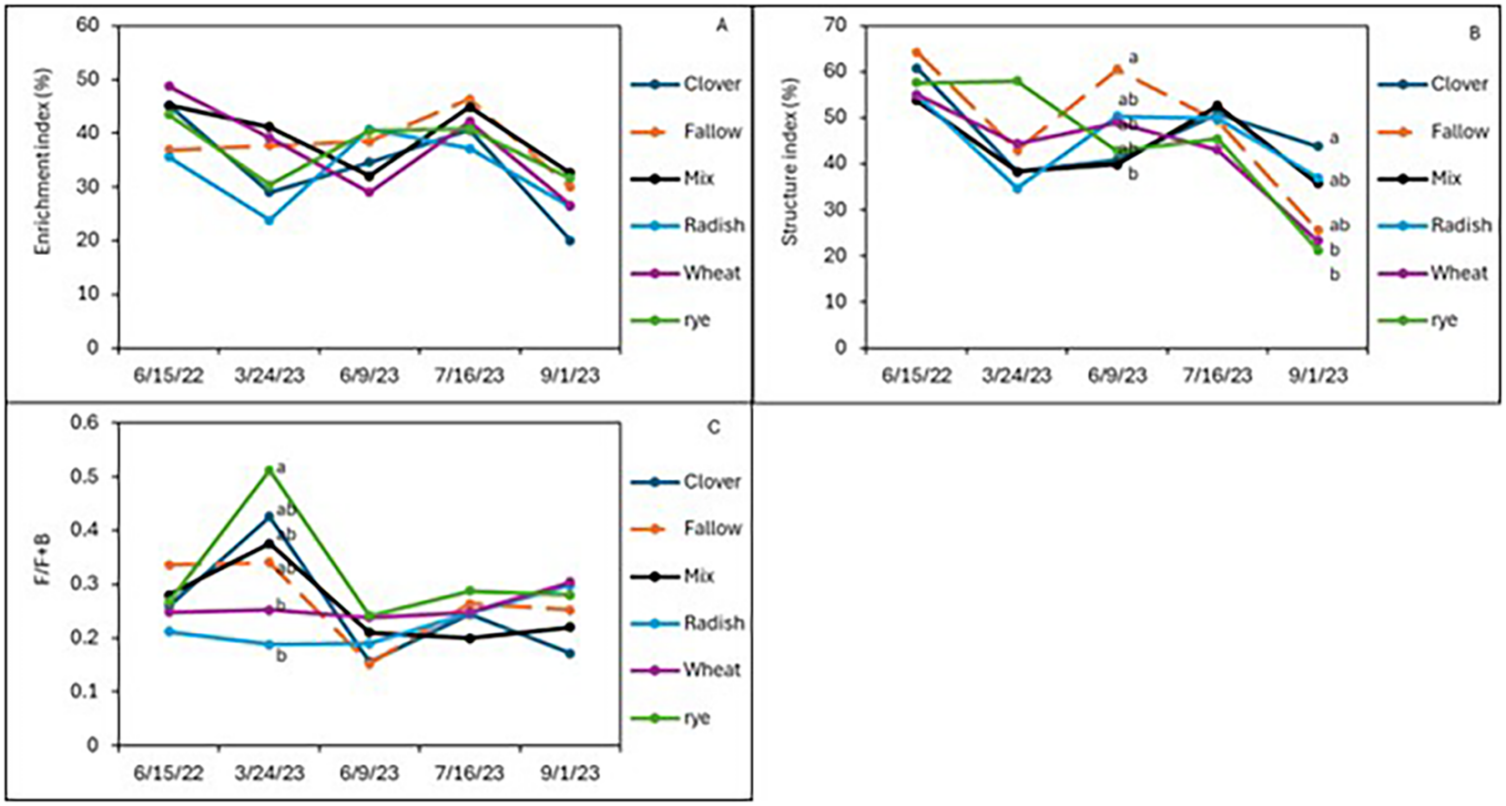
Soil nematode community indices of enrichment **(A)**, structure index **(B)**, and channel index **(C)** from sweet potatoes following the winter cover crops in 2023.

**Figure 5:**
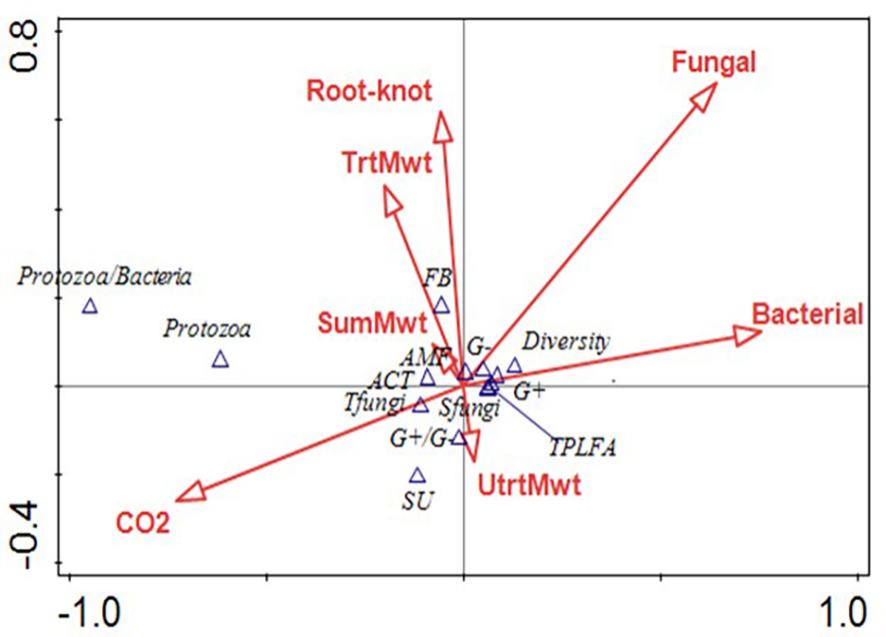
Canonical analysis of variance showing the relationships between bacterivorous nematodes (Bac), fungivorous nematodes (Fungi), herbivorous nematodes (Herb), *Meloidogyne incognita* nematodes (Root-knot), predatory nematodes (Pred), Solvita CO_2_ Burst measurement (CO_2_), nematode channel index (CI), sweet potato yield (Yield), nematode structure index (SI), omnivorous nematodes (Omni), nematode diversity (Diver), nematode enrichment index (EI), and nematode genera richness (Rich) on the arrows and arbuscular mycorrhizal fungi (AMF), actinomycetes (ACT), Gram-positive bacteria (GramP), total bacteria (Tbact), fungi:bacteria ratio (FB), Gram-negative bacteria (GramN), total fungi (Tfungi), saprophytic fungi (SFungi), ratio of saturated to unsaturated bacteria (SU), ratio of Gram-positive to Gram-negative bacteria (GPGN), and total phospholipid fatty acids (TPLFA) on the points following the sweet potato season in 2022.

**Figure 6:**
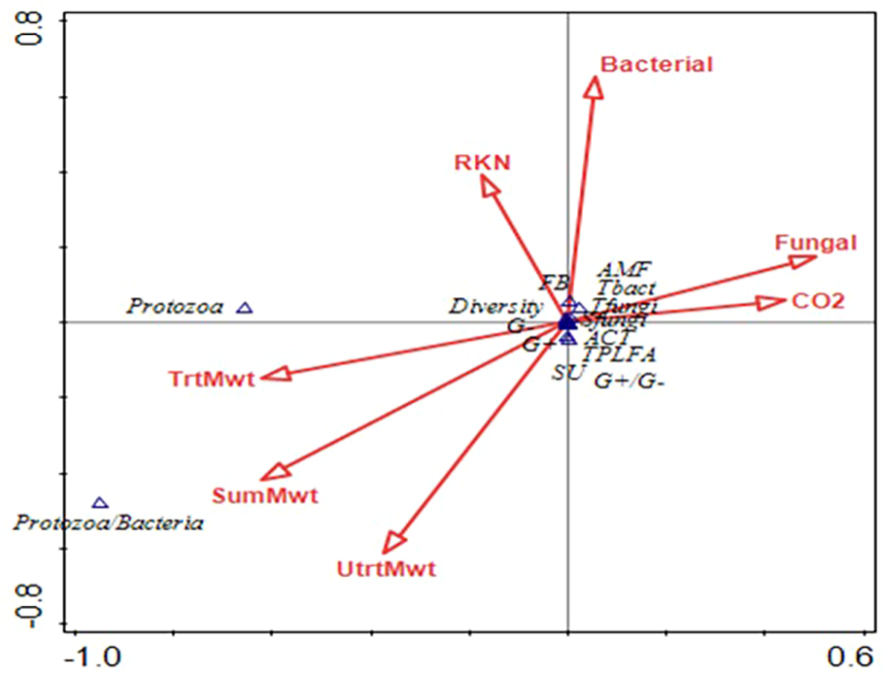
Canonical analysis of variance showing the relationships between bacterivorous nematodes (Bac), fungivorous nematodes (Fungi), herbivorous nematodes (Herb), *Meloidogyne incognita* nematodes (Root-knot), predatory nematodes (Pred), Solvita CO_2_ Burst measurement (CO_2_), nematode channel index (CI), sweet potato yield (Yield), nematode structure index (SI), omnivorous nematodes (Omni), nematode diversity (Diver), nematode enrichment index (EI), and nematode genera richness (Rich) on the arrows and arbuscular mycorrhizal fungi (AMF), actinomycetes (ACT), Gram-positive bacteria (GramP), total bacteria (Tbact), fungi:bacteria ratio (FB), Gram-negative bacteria (GramN), total fungi (Tfungi), saprophytic fungi (SFungi), ratio of saturated to unsaturated bacteria (SU), ratio of Gram-positive to Gram-negative bacteria (GPGN), and total phospholipid fatty acids (TPLFA) on the points following the sweet potato season in 2023.

At sweet potato planting, which was 3 weeks after termination of the first year of winter cover crops, microbial biomass represented by total phospholipid fatty acids (TPLFA) was similar across all cover crops. Although not statistically significant, the presence of the cover crops, averaged over all cover crops, compared to the weed fallow increased the microbial biomass by 12% with rye supporting the largest increase of 19% (**Table 5**). Samples taken during the sweet potato crop season at 30 and 84 DAP found that microbial biomass had increased by 29% and 62%, respectively, over all cover crops. Actinomycetes were most abundant in the fallow plots at 84 DAP compared to the radish, rye, and wheat cover crops. All other soil parameters and ratios were similar at the end of the first sweet potato crop year. At sweet potato planting in 2023, following the second year of winter cover crops, the relative abundance of bacteria, particularly Gram-negative bacteria, was higher following rye as compared to wheat (**Table 6**). The 30 DAP samples found that the abundance of Gram-negative bacteria and saprophytes had increased in the rye as compared to the fallow, while all other cover crops had similar microbial populations. Microbial ratios followed the same pattern, with ratios of Gram-positive to Gram-negative bacteria (GP: GN) and saturated to unsaturated fatty acids (S/U) being larger in the fallow cover crop compared to the rye cover crop. At 84 DAP, all abundance indicators were similar across all cover crop systems. Canonical correspondence analysis (CCA) included nematode communities, soil microbial abundance, soil CO_2,_ and sweet potato yield. The first two canonical axes explained 96% of the variation in 2022. Sweet potato yield was negatively impacted by *M. incognita* populations when no biopesticides were applied during the first sweet potato season based on the canonical analysis of variance (**Figure 4**). The population density of *M. incognita* increased as sweet potato growth increased. Most microbial biomasses were not linked to yield or to *M. incognita* populations, except for the fungi to bacteria ratio (F/B), which was positively related to yield in the biopesticide-treated plots. The GP: GN was also positively related to yield in the untreated plots (**Figure 5**). In 2023, the first two canonical axes explained 95% of the variation. Sweet potato yield was no longer affected by *M. incognita* populations in either biopesticide-treated or untreated plots (**Figure 6**). In the second season, sweet potato yield was negatively affected by fungivorous nematodes (Fungi) and microbial respiration (CO_2_). The biomass of the protozoa and the protozoa to bacteria ratio were positively related to yield (**Figure 6**). There was a clear inverse relationship between populations of *M. incognita* and sweet potato yield, indicating that high populations of *M. incognita* had a very harmful effect on sweet potato yield.

**Table 5:** Effect of cover crops on soil microbial profile throughout the sweet potato season in 2022.

14 Jun 2022												
Parameters	Clovers		Fallow		Mix		Radish		Rye		Wheat	
Abundance	Mean	SE±	Mean	SE±	Mix	SE±	Mean	SE±	Mean	SE±	Mean	SE±
TPLFA (ng/g)^z^	2,896.5	244.5a^y^	2,611.8	209.4a	2,870.3	401.4a	2,893.7	201.5a	3,195.7	206.1a	2,949.3	415.4a
Bact (%)	44.7	2.1a	44	2.0a	44.4	2.3a	47	2.0a	43.4	0.3a	41.7	1.3a
GN	21.7	0.4a	22.9	0.5a	21.2	0.8a	22.3	0.3a	21.3	0.7a	21.1	0.6a
GP	18.6	0.5a	16.9	0.5a	19	0.5a	18.4	0.6a	18.1	0.6a	17.9	0.9a
Actino (%)	4.4	1.6a	4.2	1.4a	4.3	1.7a	6.3	2.1a	4	1.3a	2.7	0.1a
Fungi (%)	9.8	0.8a	9.2	0.7a	9.5	0.3a	9.6	0.4a	9.6	0.6a	9.8	0.6a
AMF (%)	4.1	0.3a	3.6	0.2a	3.9	0.3a	4	0.2a	3.8	0.4a	3.8	0.2a
Sapr (%)	5.7	0.5a	5.6	0.6a	5.6	0.2a	5.6	0.4a	5.8	0.4a	6.1	0.4a
Prot (%)	0.2	0.1a	0.1	0.0a	0.1	0.0a	0.2	0.0a	0.2	0.0a	0.1	0.1a
Ratio												
F:B	0.2	0.0a	0.2	0.0a	0.2	0	0.2	0.0a	0.2	0.0a	0.2	0.0a
GP/GN	1.4	0.1a	1.6	0.1a	1.3	0.1	1.6	0.1a	1.4	0.1a	1.3	0.1a
S/U	1.6	0.2a	1.7	0.2a	1.6	0.1	1.7	0.1a	1.6	0.2a	1.5	0.1a
M:P	34.9	4.2a	40.7	9.4a	38.9	11.8	36.2	8.7a	23.8	4.5a	26.8	2.9a
PD/PR	0	0.0a	0	0.0a	0	0	0	0.0a	0	0.0a	0	0.0a
16 Jul 2022												
Abundance												
TPLFA (ng/g)	4,046.2	346.5a	4,396.1	834.4a	4,413.2	580.6a	3,901.3	300.8a	4,067.1	794.5a	4,358.7	297.1a
Bact (%)	43.2	3.5a	44.2	3.6a	40.8	3.2a	42.4	3.5a	42.4	3.1a	42.6	3.1a
GN	22.4	1.9a	22.1	1.3a	23	1.4a	23	1.9a	22.5	0.8a	22.4	1.8a
GP	15.3	1.0a	15	0.9a	13.5	1.3a	13.8	0.8a	14.8	1.2a	14.9	1.0a
Actino (%)	5.5	2.2a	7	2.0a	4.3	1.8a	5.6	1.9a	5.2	1.8a	5.3	1.9a
Fungi (%)	7	0.2a	7.1	0.9a	7.7	1.0a	5.9	0.5a	6.3	1.0a	7.3	0.9a
AMF (%)	3.4	0.3a	3.2	0.4a	3.7	0.3a	3	0.4a	2.8	0.6a	3.9	0.4a
Sapr (%)	3.6	0.3a	3.9	0.6a	4	0.8a	2.9	0.2a	3.5	0.5a	3.4	0.5a
Prot (%)	0.1	0.0a	0.1	0.1a	0.1	0.1a	0	0.0a	0.1	0.1a	0.1	0.0a
Ratio												
F:B	0.2	0.0a	0.2	0.0a	0.2	0.0a	0.1	0.0a	0.2	0.0a	0.2	0.0a
GP/GN	1.9	0.4a	1.9	0.1a	2.1	0.2a	2.1	0.3a	1.9	0.2a	1.9	0.4a
S/U	2.4	0.2a	2.6	0.4a	2.4	0.3a	2.7	0.4a	2.6	0.4a	2.5	0.4a
M:P	50.1	14.2a	63.6	19.1a	43.2	12.9a	62.5	9.2a	63.8	21.9a	41.3	5.9a
PD/PR	0	0.0a	0	0.0a	0	0.0a	0	0.0a	0	0.0a	0	0.0a
14 Oct 2022												
Abundance												
TPLFA (ng/g)	6,928.2	798.9a	7,217.6	763.3a	8,821.3	428.6a	7,280.9	738.5a	7,843.5	909.9a	8,403.9	818.8a
Bact (%)	38.4	2.6a	38.9	2.1a	36.8	1.5a	35.4	1.3a	41.4	1.9a	36.3	0.8a
GN	18.3	1.3a	17.9	0.7a	17.2	0.7a	16	1.0a	19	1.0a	16.2	0.8a
GP	13.3	1.2a	13	1.1a	13	0.3a	13.4	0.6a	15	1.0a	13.6	0.7a
Actino (%)	6.9	0.5ab	8	0.6a	6.6	0.5ab	6	0.4b	7.4	0.4b	6.4	0.4b
Fungi (%)	9	1.2a	9.1	1.5a	10	0.7a	10.8	1.7a	9	0.7a	11.6	0.8a
AMF (%)	5.5	0.8a	3.7	0.6a	5.4	0.7a	6.1	0.9a	4.6	0.4a	5.9	0.8a
Sapr (%)	3.6	0.6a	5.4	1.0a	4.7	0.6a	4.7	0.9a	4.4	0.4a	5.7	0.5a
Prot (%)	0.3	0.1a	0.3	0.1a	0.3	0.0a	0.3	0.1a	0.3	0.1a	0.3	0.1a
Ratio												
F:B	0.2	0.0a	0.2	0.0a	0.3	0.0a	0.3	0.1a	0.2	0.0a	0.3	0.0a
GP/GN	1.9	0.1a	2	0.2a	1.8	0.1a	1.7	0.1a	1.8	0.1a	1.7	0.2a
S/U	2.9	0.3a	3	0.4a	2.7	0.1a	2.7	0.2a	2.5	0.1a	2.4	0.2a
M:P	33.1	9.6a	17.6	1.7a	21.2	4.1a	24.4	4.8a	22.6	2.0a	19.8	4.1a
PD/PR	0	0.0a	0	0.0a	0	0.0a	0	0.0a	0	0.0a	0	0.0a

^y^Means ± standard error (*n* = 4) followed by the same letter(s) in a row are not significantly different at *p* ≤ 0.1 as determined using the Tukey–Kramer method. ^z^TPLFA, total phospholipid fatty acid representing total microbial biomass in nanomoles per gram of soil; Microbial groups BACT, total bacteria; GN, Gram-negative bacteria; GP, Gram-positive bacteria; AMF, arbuscular mycorrhizal fungi; ACT, actinomycetes; Fungi, total fungi; AMF, arbuscular mycorrhizal fungi; SAPR, saprophytic fungi; PROT, total protozoa; GP/GN, ratio of Gram-positive to Gram-negative bacteria; F/B, ratio of fungi to bacteria; S/U, saturated to unsaturated fatty acids; M:P, ratio of monounsaturated to polyunsaturated fatty acids; PD/PR, ratio of predator to prey.

**Table 6:** Effect of cover crops on soil microbial profile throughout the sweet potato season in 2023.

9 Jun 2023												
Parameters	Clovers		Fallow		Mix		Radish		Rye		Wheat	
Abundance	Mean	SE±	Mean	SE±	Mix	SE±	Mean	SE±	Mean	SE±	Mean	SE±
TPLFA (ng/g)	1,830.4	437.8a	1,747.7	190.1a	2,542.3	638.4a	1,966.9	289.1a	1,432.2	301.9a	1,492.3	212.0a
Bact (%)	41.8	1.0a	42.9	1.1a	39.2	1.9a	42.4	2.0a	45.5	2.7a	34.7	2.3a
GN	22	0.8a	24	0.9a	19.9	1.4a	23.2	0.8a	25.1	0.9a	19.6	1.9a
GP	13.1	0.7a	11.6	0.9a	13.1	1.3a	11.9	0.9a	12.4	1.4a	9	0.8a
Actino (%)	6.7	0.4a	7.3	0.5a	6.2	0.4a	7.3	0.4a	8	0.6a	6.1	0.4a
Fungi (%)	6.9	1.3a	6.5	0.7a	8.7	1.3a	6.8	0.6a	6.5	0.4a	6.9	1.3a
AMF (%)	4	1.2a	3.1	0.2a	5.4	0.9a	3.7	0.4a	4.2	0.5a	5	1.2a
Sapr (%)	2.9	0.3a	3.4	0.7a	3.3	0.5a	3.1	0.2a	2.3	0.2a	1.9	0.3a
Prot (%)	0	0.5a	0.4	0.4a	0	0.0a	0	0.0a	0	0.0a	0	0.0a
Ratio												
F:B	0.2	0.0a	0.2	0.0a	0.2	0	0.2	0.0a	0.1	0.0a	0.2	0.0a
GP/GN	2.2	0.2a	2.8	0.4a	2.1	0.2	2.6	0.1a	2.8	0.2a	3	0.4a
S/U	3.6	0.4a	4	0.7a	3.2	0.4	3.5	0.2a	3.8	0.3a	4.6	0.8a
M:P	63.4	17.0a	43.7	15.8a	57.2	13.2	48.5	22.8a	87.5	12.6a	87.2	12.3a
PD/PR	0	0.0a	0	0.0a	0	0	0	0.0a	0	0.0a	0	0.0a
10 Jul 2023												
Abundance												
TPLFA (ng/g)	3,211.9	467.1a	2,398.8	174.2a	3,532.9	152.1a	2,399.3	445.9a	3,297.5	431.3a	3,824.9	454.8a
Bact (%)	33.6	1.1a	33.6	4.2a	33.3	1.7a	36.8	2.9a	36.9	2.1a	33.1	1.2a
GN	18.3	0.4	19.2	2.4a	18.4	0.7a	20.4	1.4a	18.6	1.4a	17.6	0.8a
GP	9.5	1.0a	8	1.1b	9.2	1.0a	9.8	1.3ab	12.3	1.3a	10.2	0.4a
Actino (%)	5.8	0.1a	6.4	1.0a	5.7	0.3a	6.6	0.5a	6	0.4a	5.3	0.3a
Fungi (%)	6.3	0.8a	4.1	0.4a	5.5	0.7a	4.7	0.5a	6.3	0.5a	6.4	0.8a
AMF (%)	3.9	0.6a	2.3	0.3a	3.3	0.4a	2.5	0.3a	3.5	0.4a	3.9	0.7a
Sapr (%)	2.4	0.3a	1.7	0.2b	2.2	0.3a	2.1	0.3ab	2.9	0.2a	2.6	0.2a
Prot (%)	0	0.0a	0	0.0a	0	0.0a	0	0.0a	0	0.0a	0	0.0a
Ratio												
F:B	0.2	0.0a	0.1	0.0a	0.2	0.0a	0.1	0.0a	0.2	0.0a	0.2	0.0a
GP/GN	2.7	0.3a	3.3	0.3a	2.8	0.4a	2.9	0.4ab	2.1	0.2a	2.3	0.1a
S/U	4.7	0.8a	6.8	1.1a	5	0.7a	5.4	0.7ab	3.9	0.4a	4.3	0.3a
M:P	35.2	3.8a	75.1	15.9a	47.6	8.9a	51.8	14.8a	52	16.7a	48.2	10.2a
PD/PR	0	0.0a	0	0.0a	0	0.0a	0	0.0a	0	0.0a	0	0.0a
14 Oct 2023												
Abundance												
TPLFA (ng/g)	2518	389.3a	2,253.5	179.1a	1,826.1	472.3a	1,770.3	426.1a	2,114.1	298.7a	2,546.2	478.9a
Bact (%)	39.6	2.1a	39.5	2.7a	43.6	3.5a	36.2	3.4a	40.5	1.9a	42.6	3.8a
GN	19.3	2.8a	18	2.6a	22.2	1.8a	17.6	0.9a	21.3	0.9a	17.8	2.4a
GP	13.7	1.8a	15.2	2.1a	13.4	2.0a	12.2	2.5a	12	1.8a	18.7	4.6a
Actino (%)	6.6	1.0a	6.2	1.1a	8	0.8a	6.4	0.4ab	7.3	0.3a	6.1	1.0a
Fungi (%)	10	4.3a	9.1	3.0a	6.1	1.4a	6.7	0.7a	7.6	1.4a	6.6	1.5a
AMF (%)	2.8	0.3a	3.1	1.1a	3.4	0.9a	3.2	0.5a	4.4	0.8a	2.6	1.1a
Sapr (%)	7.2	4.4a	6	2.7a	2.7	0.6a	3.5	0.7a	3.2	0.6a	4	1.1a
Prot (%)	0	0.0a	0	0.0a	0	0.0a	0	0.0a	0	0.0a	0	0.0a
Ratio												
F:B	0.3	0.1a	0.2	0.1a	0.1	0.0a	0.2	0.0a	0.2	0.0a	0.1	0.0a
GP/GN	2.1	0.4a	1.8	0.4a	2.5	0.4a	2.2	0.3a	2.6	0.4a	1.8	0.6a
S/U	3.3	0.6a	3.2	0.7a	4.1	1.3a	3.6	0.6a	4	0.8a	3.1	1.0a
M:P	49.7	11.1a	55.7	13.8a	83.4	14.8a	67.9	18.2a	60.2	12.3a	169.9	72.5a
PD/PR	0	0.0a	0	0.0a	0	0.0a	0	0.0a	0	0.0a	0	0.0a

^y^Means ± standard error (*n* = 4) followed by the same letter(s) in a row are not significantly different at *p* ≤ 0.1 as determined using the Tukey–Kramer method. ^z^TPLFA, total phospholipid fatty acid representing total microbial biomass in nanomoles per gram of soil; Microbial groups BACT, total bacteria; GN, Gram-negative bacteria; GP, Gram-positive bacteria; AMF, arbuscular mycorrhizal fungi; ACT, actinomycetes; Fungi, total fungi; AMF, arbuscular mycorrhizal fungi; SAPR, saprophytic fungi; PROT, total protozoa; GP/GN, ratio of Gram-positive to Gram-negative bacteria; F/B, ratio of fungi to bacteria; S/U, saturated to unsaturated fatty acids; M:P, ratio of monounsaturated to polyunsaturated fatty acids; PD/PR, ratio of predator to prey.

## 4 Discussion

### 4.1 Nematodes

All winter cover crops supported more than 10 J2 of *M. incognita*/100 cm^3^ soil, which is considered the threshold level at planting (Becker and Westerdahl, 2016; Schloemer et al., 2025). Elbon rye and the cover crop mixture produced the highest biomass, with elbon rye and crimson clover supporting higher populations of *M. incognita* than other cover crops. Since crimson clover is a legume, it adds nitrogen to the soil, so crimson clover may be a good option for fields without nematode problems (Jackson and Harrison, 2008). Similar higher nematode populations have been observed with *Rotylenchulus reniformis* Linford and Oliveira following legume cover crops of lupins, crimson clover, and vetch (Schloemer et al., 2025). In the plots following the legume cover crop, crimson clover supported numerically elevated populations of *M. incognita* at midseason (60 DAP), emphasizing leguminous cover crops’ role in maintaining plant-parasitic nematode populations. Jackson and Harrison (2008) affirmed the value of leguminous cover crops in the organic sweet potato system, mentioning that legume cover crops could potentially fulfill sweet potatoes’ relatively low nitrogen fertilization needs. DuPont et al. (2009) found that nematode abundance was 72% higher in cover crop treatments containing legumes than in fallow, and plant productivity was positively associated with legume cover crops in year 2 of their study. Since 2022 and 2023 were relatively dry years in this study, the relative drought impacted the soil populations of *M. incognita*. At midseason (30 DAP), the population density of *M. incognita* was low across all winter cover crops, probably corresponding to the nematode life cycle with the J2 stage moving out of the soil and infecting the sweet potato roots (Moens et al., 2009). At midseason (60 DAP), the plots following crimson clover and field peas had the numerically highest populations of *M. incognita*. This suggests a link between leguminous cover crops and increased population densities of *M. incognita*, which was also highlighted by Gill et al. (2023), who emphasized that winter legumes like crimson clover and hairy vetch can increase the populations of *M. incognita*. In general, cereal winter cover crops, like elbon rye, are more effective than leguminous winter cover crops for nematode suppression (Wang et al., 2004). Elbon rye’s deleterious effect on the soil populations of *M. incognita* was observed at the pre-sweet potato harvest sampling. The plots following the elbon rye numerically supported the lowest soil *M. incognita* populations, a 63% decrease compared to the fallow.

### 4.2 Nematode community

The composition of the soil nematode community can provide valuable insights into the health of the soil ecosystem, as changes in agricultural management practices, like winter cover cropping, can influence the nematode community structure (Bongers and Bongers, 1998). However, no distinct differences in nematode community composition between winter cover crops were observed. Two growing seasons may not be enough time to detect winter cover crop-induced changes in the soil nematode community (Blanco-Canqui et al., 2015). Additionally, sweet potato production requires intense disturbance to the soil from planting (which requires hilling or bed mounding) to harvest (which requires deep digging) (Agbede and Adekiya, 2009). These soil disturbances may have overshadowed changes in the soil nematode community due to winter cover cropping (Wang et al., 2022). Overall, it was clear that upon the conclusion of 2 years of sweet potato cultivation and winter cover cropping, there were no distinct differences between the winter cover crops tested.

### 4.3 Insect damage

The primary insect pests belonged to the WDS complex (wireworm, *Diabrotica* spp., and *Systena* spp.). Across winter cover crops, damage by this pest complex was similar, although the highest was following daikon radish. This damage may be due to the similarity in root structure. The radishes may have acted as a green bridge providing food and habitat for insect pests to survive during a time that the soil is typically left fallow (Jackson and Harrison, 2008). These green bridges allow higher larval insect pest survival through the winter, which could increase the risk of damage to the following sweet potato crop (Favetti et al., 2017). A similar trend was observed with daikon radish cover cropping, leading to increased sweet potato flea beetle damage to the following sweet potato crop. The daikon radish cover crop could have provided the green bridge or winter food source for the sweet potato flea beetle in our trials, as radish produces a large root present over the winter months. The addition of biopesticides statistically reduced WDS complex and total insect damage in the sweet potatoes. We saw significantly fewer incidences of WDS damage when BotaniGard 22WP, Triple Threat Entomopathogenic Nematodes, and Majestene were applied. This is similar to Huseth et al. (2021), who found that a tank mix of Majestene and Brigade resulted in fewer WDS holes than the untreated plots.

### 4.4 Yield

A numeric sweet potato yield benefit was observed when applying the biopesticides (BotaniGard 22 WP, Triple Threat Entomopathogenic Nematodes (EPNs), and Majestene) when the sweet potatoes were challenged with *M. incognita*. Researchers at the University of Georgia also found that Majestene produced significantly higher squash yield in a field with *M. incognita* (Nnamdi et al., 2022). This finding is like that of Watson et al. (2023) in Louisiana, who observed that Majestene resulted in a higher yield of sweet potato grade No. 1 under *R. reniformis* pressure. This benefit could be due to Majestene’s plant growth-promoting effects, which have been documented with other nematicides, including aldicarb (Reddy et al., 1990). This trend toward plant growth promotion was also seen in our greenhouse test, where the plants treated with Majestene had a 6% greater biomass compared with the untreated control (Schloemer et al., in review). Entomopathogenic nematodes have also had yield-enhancing effects. In cotton, the application of EPNs *S. carpocapsae* and *H. bacteriophora* resulted in increased cotton yield compared to the untreated control (Nagachandrabose, 2012).

### 4.5 Soil health

The Solvita CO_2_ respiration test is a simple and quick method to quantify microbial activity in soils and track the results of management changes (Haney et al., 2008). Higher CO_2_ release is related to the amount or quality of organic carbon and nitrogen in the soil and is considered an indicator of biological attributes linked to healthy soil functioning (Haney et al., 2008). Chahal and Van Eerd (2019) found that Solvita CO_2_ burst values were the lowest with a no cover crop treatment compared with oilseed radish and rye, which supports our findings that the fallow was consistently among the lowest treatments for soil respiration. Blanco-Canqui et al. (2015) also emphasized the increase in soil microbial activity with the use of cover crops. This agrees with our findings, where the cover crop mix (crimson clover, daikon radish, wheat, and elbon rye) numerically produced the highest microbial respiration, at sweet potato planting and near harvest, indicating that the soil was more biologically active following the mix of legume and grass cover crops. Chahal and Van Eerd (2019) also found that their cover crop mixture of oilseed radish and rye produced higher soil respiration values than either of the cover crops alone. This indicates a collaborative effect when combining winter cover crops. This collaborative effect could be due to the variety of cover crops stimulating more microbes in the soil and creating a richer environment for microbes to thrive, which relates to soil health. However, the effects of cover cropping on soil carbon concentration are often not detectable in the first few years after establishment (Blanco-Canqui et al., 2015).

Based on the canonical analysis, sweet potato yield was positively related to the ratio of Gram-positive to Gram-negative bacteria. Since the presence of Gram-negative bacteria, like *Bacillus* spp., is associated with hardy environments, this indicates that sweet potatoes yield better in a more resilient soil environment (Paudel et al., 2021). These data also suggest that sweet potato yield is enhanced when soil food web structure is less disturbed, as indicated by a high nematode structural index (Du Preez et al., 2022). The relationship between microbial respiration and high saprophytic fungal biomass suggests that the majority of microbial respiration was dominated by fungal decomposition (Paudel et al., 2021). More abundant total microbial biomass (measured as total phospholipid fatty acids) was closely related to higher nematode diversity, which shows that higher microbial biomass can support the flourishing of a variety of free-living nematodes and an increase in soil health (Paudel et al., 2021). Additionally, the analysis shows an acute inverse relationship between sweet potato yield and populations of *M. incognita*. This clearly indicates the importance of managing plant-parasitic nematode populations to achieve high sweet potato yields, as emphasized by Ploeg et al. (2019).

## 5 Conclusions

The objective of the field study was to evaluate sweet potato yield quality and quantity following winter cover crops and biopesticides for the management of *M. incognita* and insect pests while assessing the impacts on soil health and the nematode community. Our findings indicate that the combination of BotaniGard 22WP, Triple Threat Entomopathogenic Nematodes, and Majestene significantly reduced insect damage to sweet potatoes under field conditions. The winter cover crops, elbon rye, and the mix containing crimson clover, daikon radish, elbon rye, and wheat resulted in lowered soil *M. incognita* populations when compared with leguminous winter cover crop crimson clover. Total insect pest damage was similar across winter cover crops, but the lowest was following crimson clover. Soil health values measured by the Solvita CO_2_ Burst test were elevated following the winter cover crop mixes compared with the single winter cover crop treatments, indicating that the mixes stimulate higher maximal biological activity, which relates to soil health. Improvements in soil health due to cover crops and biopesticides found reduced effects of *M. incognita* on sweet potato yields over time.

## 6 Management summary

This field study showed that integrating winter cover crops with biopesticides can improve sweet potato production while managing pests and supporting soil health. The combined use of *BotaniGard 22WP*, Triple Threat Entomopathogenic Nematodes, and *Majestene* effectively reduced insect damage to roots. Among cover crops, elbon rye and a multi-species mix (crimson clover, daikon radish, elbon rye, and wheat) suppressed *M. incognita* populations more effectively than crimson clover alone. Multi-species cover crop mixes also enhanced soil health, as shown by higher biological activity, compared with single-species cover crops. Together, these practices reduced nematode impacts on yield and improved sweet potato quality over the 2-year study.

## 7 Limitations and future work

While these results demonstrate clear benefits of integrating cover crops and biopesticides, the study was limited to a single location and set of seasonal conditions, which may affect the generalizability of the findings. In addition, only a subset of commercially available biopesticides and cover crop species were tested. Future work should evaluate these strategies across multiple environments, soil types, and management systems to confirm the consistency of outcomes. Longer-term studies are also needed to assess the cumulative impacts on nematode communities, soil health indicators beyond CO_2_ respiration, and the economic feasibility of adopting these integrated practices at commercial scales.

## Data Availability

The original contributions presented in the study are included in the article/**Supplementary Material**. Further inquiries can be directed to the corresponding author.
